# Supplementary material for: Comparing the Differences in Adverse Events among Chimeric Antigen Receptor T-Cell Therapies: A Real-World Pharmacovigilance Study
Source: Pharmaceuticals (Basel). 2024 Aug 5;17(8):1025. doi: 10.3390/ph17081025 (PMC11359317; doi:10.3390/ph17081025)
Supplement: Supplementary file 1 [file pharmaceuticals-17-01025-s001.zip › pharmaceuticals-3122706-supplementary.pdf]

**Supplementary Materials:** Table S1: Two-by-two contingency table for disproportionality analysis.

| | Target event | Other events | Sums |
|---|---|---|---|
| Target drug | a | b | a+b |
| Other drugs | c | d | c+d |
| Sums | a+c | b+d | a+b+c+d |

ROR=$(a/c)/(b/d)$; ROR 95% CI=$e^{\ln(ROR)\pm1.96\sqrt{\left(\frac{1}{a}+\frac{1}{b}+\frac{1}{c}+\frac{1}{d}\right)}}$; PRR=$\frac{a/(a+b)}{c/(c+d)}$; $\chi^2 = \frac{(|ad-bc|-\mathrm{N}/2)^2\mathrm{N}}{(a+b)(a+c)(c+d)(b+d)}$
